# Polyethylene glycol-based hydrogel rectal spacers for prostate brachytherapy: a systematic review with a focus on technique

**DOI:** 10.1007/s00345-020-03414-6

**Published:** 2020-08-25

**Authors:** S. Vaggers, B. P. Rai, E. C. P. Chedgy, A. de la Taille, B. K. Somani

**Affiliations:** 1 University Hospital Southampton NHS Trust, Southampton, UK; 2 Freeman Hospital, Newcastle, UK; 3 Department of Urology, Robotic and Miniinvasive Surgery, Assistance Publique des Hopitaux de Paris, 94000 Créteil, France

**Keywords:** Prostate, Cancer, Rectal spacer, Brachytherapy, SpaceOAR, DuraSeal, Radiotherapy

## Abstract

**Introduction:**

Radiation dose to the rectum in prostate brachytherapy (PBT) can be reduced by the use of polyethylene glycol (PEG) hydrogel spacers. This reduces the rate of rectal toxicity and allows dose escalation to the prostate. Our objectives were to provide an overview of technique for injection of a PEG hydrogel spacer, reduction in rectal dosimetry, gastrointestinal toxicity and potential complications.

**Methods:**

We systematically reviewed the role of PEG hydrogel spacers in PBT using the Cochrane and PRISMA methodology for all English-language articles from January 2013 to December 2019. Data was extracted for type of radiotherapy, number of patients, type of PEG-hydrogel used, mean prostate-rectum separation, rectal dosimetry, acute and late GI toxicity, procedure-related complications and the technique used for hydrogel insertion.

**Results:**

Nine studies (671 patients and 537 controls) met our inclusion criteria. Of these 4 used DuraSeal^®^ and 5 used SpaceOAR^®^. The rectal spacing achieved varied between 7.7-16 mm. Failure of hydrogel insertion was seen only in 12 patients, mostly related to failure of hydrodissection in patients undergoing salvage PBT. Where reported, the rectal D2 cc was reduced by between 21.6 and 52.6% and the median rectal V75% cc was reduced by between 91.8–100%. Acute GI complications were mostly limited to grade 1 or 2 toxicity (*n* = 153, 33.7%) with low levels of grade 3 or 4 toxicity (*n* = 1, 0.22%). Procedure-related complications were limited to tenesmus (0.14%), rectal discomfort (1.19%), and bacterial prostatitis (0.44%).

**Conclusions:**

PEG hydrogel spacers are safe to insert. Gel insertion is easy, fast and has a low rate of failure. These studies convincingly demonstrate a significant reduction in rectal dosimetry. Although the results of spacers in reducing rectal toxicity is promising, these need to be confirmed in prospective randomised trial.

**Electronic supplementary material:**

The online version of this article (10.1007/s00345-020-03414-6) contains supplementary material, which is available to authorized users.

## Introduction

Prostate brachytherapy (PBT) is a definitive treatment for prostate cancer [1, 2]. Low-dose-rate (LDR) or high-dose-rate (HDR) PBT can be used alone or in combination external beam radiotherapy (EBRT) to treat low, intermediate and high-risk prostate cancer [3–6]. Dose escalation is strongly linked to a reduction of biochemical and clinical failure and metastasis-free survival [7]. However, the benefits of dose escalation must be balanced with the risk of increased radiation dose to the bladder, urethra and in particular, the rectum [8]. The higher the radiation dose received by the rectum the higher the risk of gastrointestinal (GI) toxicity [9, 10].

An effective way to limit the radiation exposure and toxicity to the rectum is to increase the distance between the rectum and the prostate using a spacer [11]. There are many different types of spacers including hyaluronic acid, biodegradable balloons, collagen and polyethylene glycol (PEG) hydrogel [12]. A PEG hydrogel is a hydrophilic polymer that can be cross-linked into a network which can retain a large quantity of water. Even minimal increases in the distance between the prostate and rectum significantly reduces the dose delivered to the rectum because of the rapid dose fall off with PBT.

This systematic review evaluates the space creation, rectal dosimetry, failure, and acute and late GI toxicity. Furthermore, we review the variation in techniques described in the literature and ‘Tips and tricks’ associated with it.

## Materials and methods

### PICO statement

Population-Patients with prostate cancer receiving brachytherapy.

Intervention-PEG-Hydrogel spacer, e.g., SpaceOAR or DuraSeal.

Comparison-No spacer.

Outcomes-Procedure-related complications, procedure failures, Prostate-rectum separation, rectal dosimetry and radiation-related GI toxicities (acute and late) and technique for hydrogel insertion.

### Evidence acquisition: criteria for considering studies for this review

Inclusion criteria

- Studies reporting on PBT with PEG hydrogel spacers.
- Salvage and primary treatment.

Exclusion criteria

- Low volume studies of < 10 patients.
- Case reports, review articles and editorials.
- Non-English language studies.
- Animal and laboratory studies.

### Search strategy and study selection

**Fig. 1:**
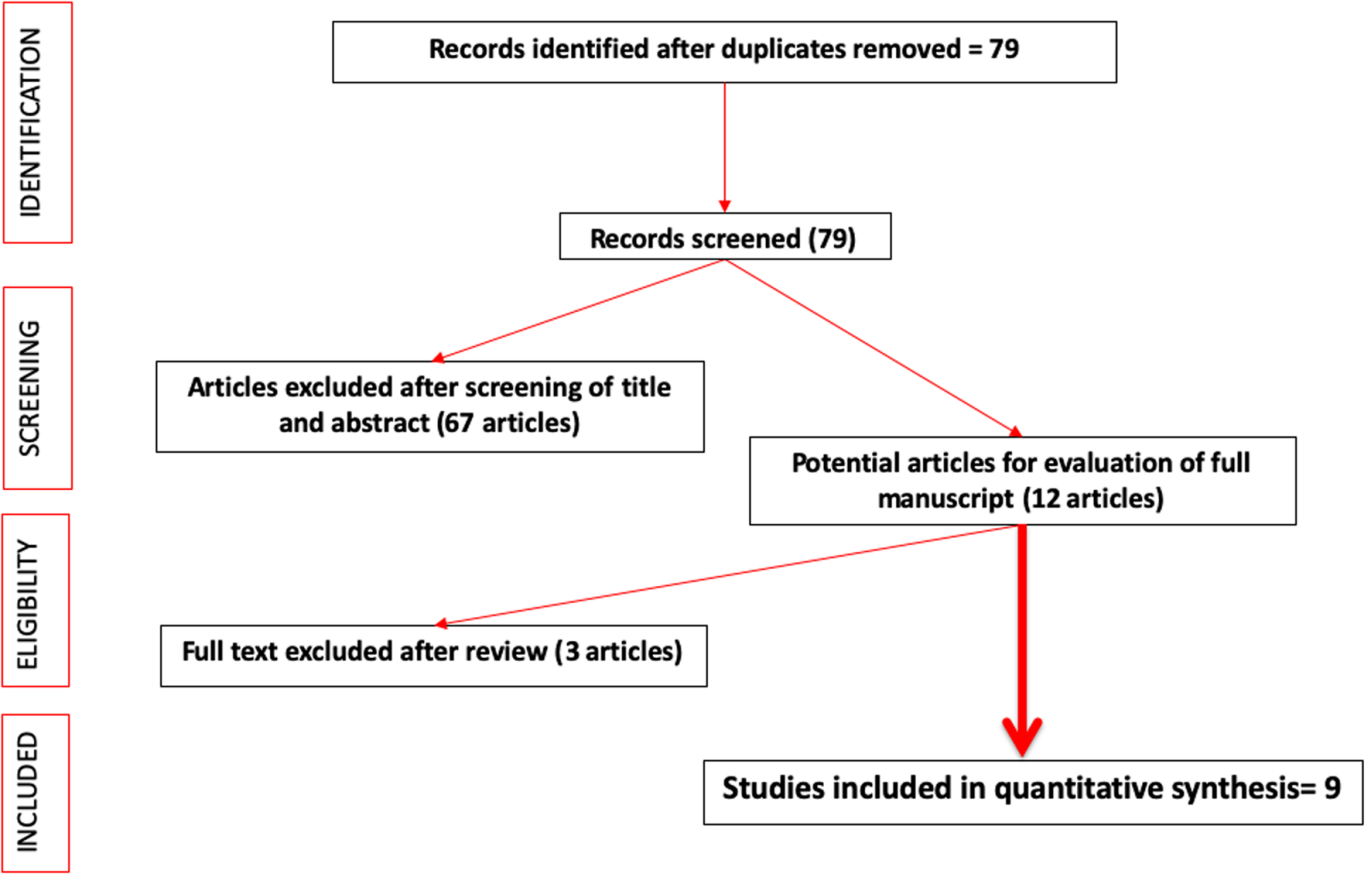
PRISMA flow diagram to demonstrate screening process for included and excluded papers

We performed a systematic review in a Cochrane style to identify all original articles relating to polyethylene hydrogel spacers for PBT. The Preferred Reporting Items for Systematic Reviews and Meta-Analyses (PRISMA) checklist was adhered to. A literature search was conducted through PubMed/MEDLINE, EMBASE, CINAHL, Cochrane library, Clinicaltrials.gov and Google Scholar and citation lists and references were also evaluated. Search terms included (not limited to) ‘Hydrogel spacer’, ‘Spacer’, ‘DuraSeal, ‘SpaceOAR hydrogel’ OR ‘Polyethylene glycol hydrogel’, ‘Brachytherapy’, ‘Prostate brachytherapy’, ‘low dose rate’, ‘LDR’, ‘high dose rate’, ‘HDR’, ‘rectal separation’ and ‘prostate rectal spacer’. The search was limited to English language publications between January 2013 and December 2019 (see Fig. 1). The references of included studies were checked to search for additional eligible studies.

Two reviewers (SV and BKS) identified the studies that appeared to fit the inclusion criteria based on their abstracts for a full review. Studies of more than ten patients were fully reviewed. Studies of less than ten patients and case reports were reviewed only for procedure related complications. Data was then extracted including type of radiotherapy, number of patients, type of PEG-hydrogel used, mean prostate-rectum separation, rectal dosimetry, acute and late GI toxicity, other procedure-related complications and the technique used for hydrogel insertion. However, not all papers used RTOG guidelines to grade complications. We have not included genitourinary complications as hydrogel spacers do not reduce the dose delivered to the urethra [13]. The heterogeneity of available evidence did not allow for formal meta-analysis to be performed.

## Results

### Search results

After removing duplicates, 79 articles were identified. These abstracts were screened, 12 full text articles were reviewed and 9 were included in the final review (see Fig. 1). In total, 671 patients received a PEG spacer alongside PBT as either a salvage or definitive treatment with 537 controls who did not have a PEG spacer. All papers were retrospective case series published within the last 5 years, six of the papers included controls. Characteristics and results of the studies included are summarised in Table 1. A further four articles of case reports or < 10 cases patients receiving PBT were reviewed for procedure-related complications.

**Table 1:** Summary of characteristics and results of studies included in the systematic review including treatment technique, spacer type, mean separation and acute and late GI complications

First author (year)	Treatment technique	No. of patients	Spacer type	Median follow up time	Mean separation (mm)	Rectal dosimetric reduction (spacer vs. non spacer)/ (percentage dose reduction)	Complication scoring system	Acute GI toxicity (spacer vs. non spacer)	Late GI toxicity (spacer vs. non spacer)	Failure rate
Mahal et al. [14]	Salvage LDR-BT; prior pelvic irradiation	11	DuraSeal	15.7	10.9 in patients with prior BT and 7.7 in patients with prior EBRT	Median V75% (cc): 0.07	EPIC-CP questionnaire	Grade 1: 0% Grade 2: 9% (fistula)	Grade 1 or 2: 36% Grade 3 or 4: 9% 1 case of prostatorectal fistula requiring a diverting colostomy and an interposition rotational gracilis muscle flap	27.2%
Heikkilä et al. [15]	LDR-BT	10	DuraSeal	–	10	Rectal D2 cc 64 ± 13 Gy vs. 95 ± 13 Gy (*p* = 0.005)/(32.6%)	–	One patient reported a sensation of pressure in the rectum One patient felt a sudden need for defecation	–	0%
Wu et al. [16]	HDR-BT; HDR-BT + EBRT; Salvage HDR-BT	18 with gel; 36 without gel	SpaceOAR	–	–	Median V75% (cc): < 0.005 vs. 0.12 (*p* ≤ 0.0005)/(100%)	–	One patient developed a rectal abscess	–	0%
Chao et al. [17]	HDR-BT + EBRT	30 with gel; 65 without gel	SpaceOAR	58	–	Median V75% (cc) 0.0 (0–0.22) vs. 0.45 (0–1.46) (*p* ≤ 0.001)/(100%)	NCICTCAE v4.0	Grade 1: 13.3% vs. 30.8% (*p* = 0.05) Grade 2 0% vs. 1.5% (*p* = 0.48)	Grade 1 0% vs. 7.7% (*p* = 0.11)	–
Chao et al. [18]	HDR-BT + EBRT	32 with gel and 65 without gel	SpaceOAR	60	10	Median V75% (cc) 0.0 vs. 0.45 (*p* ≤ 0.001)/(100%)	NCICTCAE v4.0	Grade 1 12.5% vs. 30.8% (*p* = 0.05)	Grade 1: 0% vs. 7.7% (*p* = 0.11)	–
Strom et al. 2014 [19]	HDR-BT ± IMRT	100 with gel; 100 without gel	DuraSeal	8.7	12	Rectal D2 cc 47 ± 9% vs. 60 ± 8% (*p* < 0.001)/(21.6%)	–	–	–	0%
Yeh et al. [20]	HDR-BT + IMRT	326	DuraSeal	16	16	Maximum dose to rectum 78% vs. 95% (SD = 11.9%)/(17.3%)	NCICTCAE v4.0	Grade 1: 37.4% Grade 2: 2.8% Most commonly diarrhoea	Grade 1:12.7% Grade 2: 1.4% Grade 3: 0.7% 1 case of severe proctitis One case of fistula and necrotising fasciitis requiring a diverting colostomy	–
Taggar et al. [13]	LDR-BT; LDR-BT + EBRT; Salvage LDR-BT	74 with gel; 136 without gel	SpaceOAR	6	11.2	Rectal D2 cc 20.47% vs. 43.16% (*p* = 0.000)/(52.6%)	RTOG	Diarrhoea: LDR alone 7.7% vs. 15.9% LDR + EBRT 12.5% vs. 4.1% Salvage 12.5% vs. 5.3% Proctitis: LDR alone 0% vs. 0%, LDR + EBRT 0% vs. 5.5% Salvage 0% vs. 0% No grade ¾ complications	–	6.8%
Morita et al. [21]	LDR-BT; LDR-BT + ERBT	100 with gel; 200 without gel	SpaceOAR	–	11.6	Median V100% 0.026 ± 0.14 vs. 0.318 + /1 0.34 (*p* ≤ 0.001)/(91.8%)	–	–	–	4%

*LDR* low dose rate; *HDR* high dose rate; *BT* brachytherapy; *ERBT* external beam radiotherapy; *IMRT* intensity modulated radiotherapy; *VMAT* volumetric modulated radiotherapy; *GI* gastrointestinal; *EPIC-CP* Expanded Prostate Cancer Index Composite for Clinical Practice; *RTOG* Radiation Therapy Oncology Group; National Cancer Institute Common Terminology Criteria for Adverse Events v. 4.0 grading system

### Rectal spacing device

Four studies used DuraSeal Spinal Sealant System (Covidien, Mansfield, MA) and 5 used SpaceOAR (Augmenix, Waltham, MA). Since 2017 all the studies have used SpaceOAR and prior to this they all used DuraSeal. Only 2 papers commented on the clearance of the PEG spacer. One study using DuraSeal noted in 80% of patients the spacer was fully resorbed by week 4 [20]. Another study analysed the clearance of DuraSeal and found that despite a gel volume clearance half-life of 47 days the rectal spacing remained longer with a half-life of 110 days due to localised oedema [15].

### Rectal spacer insertion technique

**Fig. 2:**
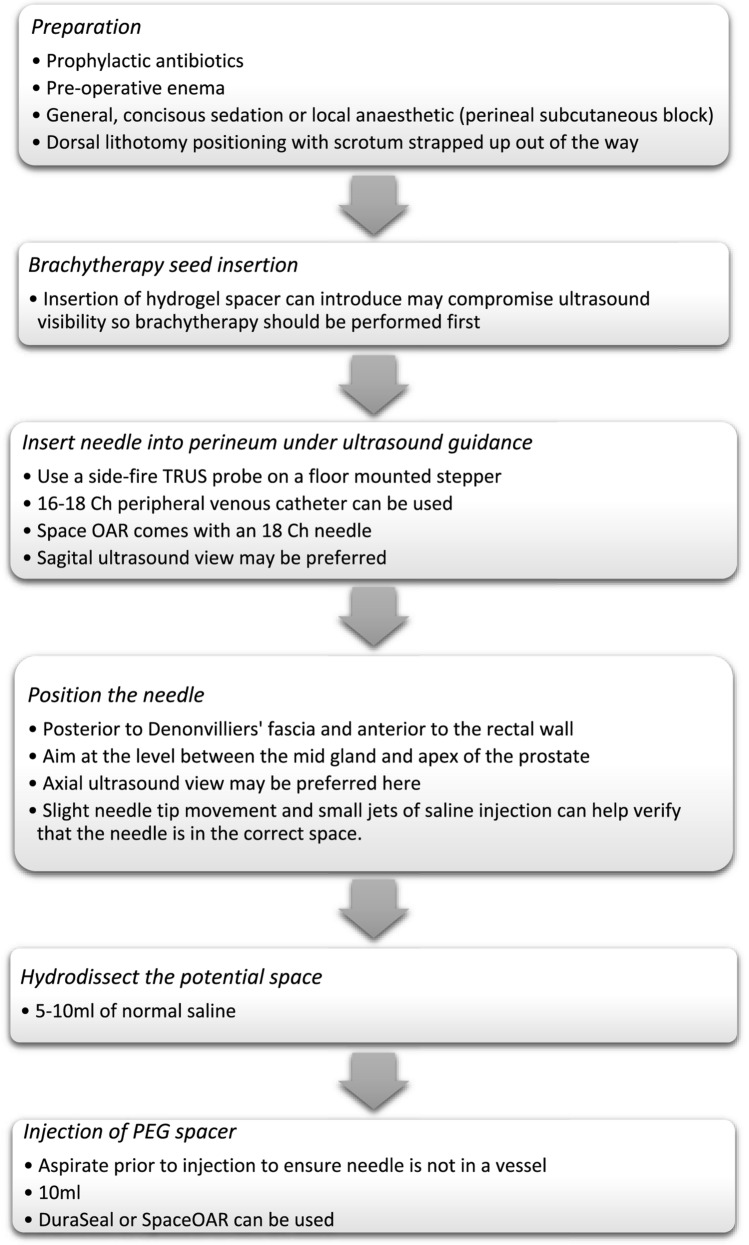
Flow diagram to demonstrate steps for insertion of PEG hydrogel spacers

Figure 2 provides a summary of rectal spacer insertion technique. Antibiotic prophylaxis was mentioned in two of the nine articles. One centre gave 10 days of 500 mg oral ciprofloxacin twice a day along with intraoperative gentamicin 80 mg and cefazolin 1 g [20]. Another centre adjusted their antibiotic prophylaxis, initially giving two doses of 500 mg oral ciprofloxacin, and then switching to one dose of intravenously ceftriaxone 1 g and gentamicin 1.5 mg/kg 30 min prior to the procedure [19]. Two studies gave patients an enema preoperatively [16, 20].

As for patient positioning, the studies used a dorsal lithotomy position. In all articles the PBT procedure was performed first. In all but one case the PEG spacer was inserted immediately after this. In one study, where two HDR PBT treatments were given a week apart, the spacer was injected during the second implant [20]. All procedures using the SpaceOAR kit used the 18-gauge needle provided, otherwise a 16 to 18-gauge peripheral venous catheter was used. The tracking needle was then removed, and the plastic catheter was left in situ.

The needles were inserted into the perineum under ultrasound (USS) guidance. The use of a floor-mounted stepper freed both of the physician’s hands for the rest of the procedure. A number of studies specifically refer to use of the sagittal view [15, 19, 21]. All of the studies reported positioning the needle posterior to Denonvilliers' fascia and anterior to the rectal wall, usually aiming at the level between the mid gland and apex of the prostate [16, 18, 21]. Most of the articles used the axial view for guiding gel placement [13, 14, 19]. All but one [20] article then hydrodissected the potential space using 5–10 ml of normal saline. Following this, 10 ml of PEG spacer was injected into the same space. The SpaceOAR comprised of two liquids including a precursor and an accelerator which was mixed during injection and polymerised over 8–10 s. Two studies using DuraSeal diluted the substance 1:1 with saline prior to injection [14, 15]. There was a degree of variation in the mean separation achieved, notably lower in salvage cases [13, 14]. One study recorded the median time of the placement of Space OAR and found it to be 4.1 min (range 3.1–12.5 min) which included including the preparation time of the applicator kit [21].

### Prostate-rectum separation

Rectal spacing was analysed in 6 out of the 9 papers, using CT or T2 weighted MRI scan. The lowest spacing achieved was 7.7 mm in patients with previous EBRT. In the other groups spacing was between 10–16 mm. These spacings cannot be directly compared as a variety of techniques were used to measure the spacing distance with most studies measuring the largest distance between the posterior edge of the prostate and anterior edge of the rectum [14, 20, 21]. Some studies measured the separation at the midgland [13, 18], which is usually the point of greatest separation [22]. One study used the mean of 3 transversal slices along the US probe, in the middle and from the 0.5 cm from the apex and base [15]. BMI was shown not to affect the rectal spacing achieved. A study of 100 patients found that the DuraSeal gel significantly increased the mean prostate–rectal distances and decreased rectal radiation doses, regardless of BMI [19].

### Failure

Although several studies reported 100% success with PEG hydrogel insertion, there were also failures reported in 12 patients across the studies. The most common reason (*n* = 9) was due to failure of hydrodissection in patients undergoing salvage PBT [13, 14, 21], the procedure was aborted if there was significant resistance at this stage. Two procedures were aborted due to unsuccessful hydrodissection of an unknown cause [13] and 1 due to operator inexperience due to premature coagulation of the solution during injection [21]. Table 2 summarises a number of tips which can be used to overcome problems with PEG hydrogel insertion.

**Table 2:** Tips and tricks of overcoming failure with the procedure

Failure	Overcoming the problem
Air bubbles	Remove all air bubbles from the endocavity balloon before starting Prime the needle with saline [23]
Premature coagulation/ needle plugging	DuraSeal can be diluted 1:1 in normal saline to reduce the speed of coagulation [24] Do not prime the SpaceOAR applicator [23] Inject SpaceOAR in one continuous movement [23]
Failure to hydrodissect	Caution in patients with prior radiotherapy, TURP, cryotherapy or prostatectomy [14] Start with small 1 ml injections to confirm in the correct place and ease of dissection [23] If significant resistance, abandon procedure [14]
Unfamiliarity with the procedure	This procedure requires familiarity with training and experience in transperineal interventional procedures Trial this with patients under general anaesthetic [11]

### Acute and late GI toxicity

Only 3 papers [13, 17, 18] compared radiation-related GI toxicity in a spacer and non-spacer group. Two of these papers reported on the same patient group [17, 18] receiving HDR PBT with EBRT. They found a significantly lower rate of grade 1 acute GI complications with 12.5% in the spacer group and 30.8% in the control group (*p* = 0.05) but no statistically significant different in grade 2 acute GI complications with 0% in the spacer group and 1.5% in the control group (*p* = 0.48). There was less late grade 1 GI toxicity (more than 3 months after finishing treatment), 0% in the spacer group compared to 7.7% for non-spacer group although this was not statistically significant. No late grade 2 or 3 GI toxicities were observed.

The other case–control study contained patients receiving LDR PBT and only reported acute toxicity. This study reported a 6 month grade 1 or 2 toxicity in 20.3% and 24.3% in patients with spacer and non-spacer groups, respectively (*p* = 0.95) [13]. Rectal discomfort was noted in 8.1% of patients with spacers but in none of the patients without spacers.

### Procedure-related complications

In addition to the studies included, 4 studies of < 10 cases [25–28] were reviewed for procedure-related complications with 2 containing procedure-related complications [25, 26]. A case study of SpaceOAR hydrogel insertion with LDR PBT reported development of a rectal ulcer 1 month after insertion. A low fibre diet was recommended, and the ulcer resolved without further intervention [25].

A report of 5 cases noted that 3/5 patients reported perineal pain or rectal discomfort, which resolved without intervention within 1 week [26]. Heikkilä et al*.* in one of the primary studies with 10 patients found that one patient reported an increased sensation of pressure in the rectum and another felt a sudden need for defecation, but both these symptoms had resolved by 3 months [15]. These complications were self-limiting and at most required over the counter medication.

Strom et al*.* reported a 6% rate of infection (bacterial prostatitis and epididymitis) in the first half of the study of hydrogel spacer in patients undergoing HDR PBT with IMRT despite patients receiving two doses of 500 mg oral ciprofloxacin [19]. With advice from an infectious disease specialist they adjusted their antibiotic prophylaxis to one dose of intravenously ceftriaxone 1 g and gentamicin 1.5 mg/kg 30 min prior to the procedure and no further infections were observed. In another study a patient receiving HDR PBT boost to EBRT developed a perineal abscess approximately 1 month after SpaceOAR insertion. This required incision, drainage and antibiotics [16].

### Dosimetry

All of the included studies reported a reduction of rectal dosimetry. In a non-randomised controlled trial of HDR-BT with or without IMRT, Strom and colleagues reported a significantly reduced rectal D_2_ from 60% without a PEG spacers compared to 47% with a PEG spacer [19]. In studies using HDR-BT (with or without EBRT), Wu et al*.* and Chao et al*.* both found significant relative reductions in rectal V50 to V80 whether in absolute risk or as a percentage of the organ at risk (OAR) [16, 18]. Chao et al*.* also found that 100% of the patients with a PEG spacer met their rectal V75 constraint, whereas only 93.8% of the patients without a PEG spacer met this requirement. In a study of PEG spacers in LDR-BT, Morita et al*.* found that the mean rectum V100 was significantly lower (0.026 cc) in the spacer group compared to the non-spacer group (0.318 cc) (*p* ≤ 0.001) [21]. A further non-controlled study in LDR-BT noted a mean rectal dose of 95 Gy (SD = 13) prior to spacer insertion and 64 Gy (SD = 13) after the spacer insertion [15]. No study found a reduction in dose to the prostate in the spacer group vs controls [14, 19–21].

All of the included studies reported an improvement in rectal dosimetric outcomes with the use of a PEG spacer. A non-randomised controlled study by Strom et al. involved 200 patients treated with HDR BT ± IMRT, half of whom were treated with PEG hydrogel prior to each brachytherapy fraction [19]. Patients with low and favourable intermediate risk disease were treated with HDR brachytherapy as monotherapy to a dose of 27–28 Gy in two fractions delivered 2–3 weeks apart. Unfavourable intermediate and high risk patients received combination treatment with IMRT, total dose of 45 Gy in 25 fractions, and HDR brachytherapy boost consisting of two 9.5–11.5 Gy fractions. The authors noted a significant decrease in the mean rectal D2_ml_ (expressed as a percentage of the prescription dose) in the spacer group (47 ± 9%) compared to the non-spacer group (60 ± 8%), (*p* < 0.001). This was regardless of patient BMI.

A retrospective study by Chao et al. reported dosimetric outcomes for patients treated with combination HDR + EBRT between 2010 to 2017 [18]. Of these, 32 patients were treated with hydrogel spacer compared to the immediately preceding 65 patients without hydrogel spacer insertion. HDR- BT (initially at a dose of 18 Gy in 3 fractions, subsequently 16 Gy in 2 fractions following a change in departmental protocol), was followed by EBRT within 2 weeks using IMRT (50.4 Gy in 28 fractions). The results of this study showed that there was a significant decrease in radiation dose to the rectum throughout all rectal volumes, including expressed as an absolute volume (cc) or as a percentage of the contoured organ at risk. This was more marked from rV_60_–rV_80,_ with ≥ 95% relative reduction in dose. Rectal OAR constraints based on Radiation Therapy Oncology Group Protocol 0321 (rV_75_ < 1 cc) were met in 100% of patients with a PEG spacer compared to 93.8% of patients without a PEG spacer. A further smaller study by Wu et al. of HDR BT ± EBRT showed similar findings of improved rectal dosimetric parameters across rV60-rV80 (absolute and percentage) in patients treated with SpaceOAR hydrogel [16].

LDR BT studies have also observed rectal dose sparing effects with the use of PEG spacers [15, 21]. Morita et al. looked at rectal dosimetry parameters (RV150 and RV100 on D30 CT post plan) in 100 patients undergoing LDT brachytherapy ± EBRT with SpaceOAR insertion immediately after seed implantation [21]. The control group included 200 patients previously treated with LDT BT ± EBRT without spacer insertion. Mean values ± SD for RV150 and RV100 were significantly lower (0.001 cc ± 0.00 and 0.025 cc ± 0.04, respectively) in the spacer group compared to the non-spacer group (0.026 cc ± 0.14 and 0.318 cc ± 0.34, respectively) (p < 0.001). A further non controlled study of LDR BT by Heikkilä et al. noted a reduction in mean rectal D2cc ± SD from 95 ± 13 Gy prior to gel insertion to 64 Gy ± 13 Gy after gel insertion [15]. No studies showed compromised target volume dose coverage in patients treated with spacers vs the control groups [18, 19, 21].

## Discussion

This systematic review presents the techniques, safety, and effectiveness PEG spacer insertion in PBT. We have focused on the practical considerations for injection of the PEG spacer, clinical benefits and complications associated with the procedure.

### Rectal spacer technique

There is little variation in the techniques described in the articles reviewed. All LDR or HDR PBT start with seed insertion first. On insertion of hydrogel there is the potential for bubbles to be introduced which may compromise USS visibility. When two PBT treatments were delivered a week apart, the hydrogel spacer was inserted after the second treatment, to prevent interference with the USS image during insertion of the second implant. Despite this, it was felt that the spacer still provided an important function if it was present for the second PBT and subsequent IMRT.

Using repetitive axial views and a slight needle tip movement, small jets of saline injection help to confirm accurate needle placement [23]. The gel must be injected posterior to Denonvilliers’ fascia and anterior to the anterior rectal wall to minimise the risk of pushing cancers cells away from the centre of the radiation field. Displacing Denonvilliers fascia is unlikely to cause any reduction in cancer clearance, as shown by a study of 243 prostatectomy specimens, which found that although 19% of prostate cancer invaded into Denonvilliers’ fascia, none had invaded through the full thickness of the structure [29].

Injecting the gel into the rectum has a theoretical increased risk of infection due to potential contamination of the anterior rectal wall or gel with faeces,, and therefore, some advocate abandoning the procedure if this occurs. However, in cancer centre of Irvine, Yeh et al. had a 5.5% rate of injection into the rectal lumen but there were no infections as a result of this [20]. In addition care must be taken to avoid injection directly into the rectal wall which has the potential to cause ulcers, ischaemia or increased rectal wall stress [25, 30].

Hydrodissection is a vital step in the procedure to ensure that there is a potential space to inject the gel into. If the perirectal space does not expand with saline injection, then the gel should not be injected as this would risk stress or ischaemia to the rectum. This is particularly important in salvage PBT. Prior to Mahal et al. paper there was a theoretical concern that extensive fibrosis between the prostate and the rectum would prevent the creation of a potential space and this provided a precedent for future salvage PBT [14].

### Alternatives to rectal spacers

There are numerous ways trialled to increase the space between the prostate and the rectum to attempt to reduce rectal dosimetry, including biodegradable balloons and gel spacers. Biodegradable balloons have been trialled, although it has been noted that the balloons fail technically during implantation in 4% and deflate prematurely in 11% of patients [31]. Prada et al*.* pioneered the use of a temporary gel spacer [32]. Their initial study showed that the use of a hyaluronic acid gel spacer significantly reduced the median rectal dose in IMBT or EBRT [32]. However, this technique lost popularity as further studies showed that hyaluronic acid degraded under radiation causing reduced viscosity [33]. To overcome the problems with other spacers, PEG hydrogel spacers were introduced due to their biocompatibility, uniform distribution and stability [34, 35]. In addition, a high success rate of placement was reported [30].

### SpaceOAR vs. Duraseal

The two PEG hydrogel spacers currently in use: DuraSeal and SpaceOAR have excellent biocompatibility [34]. There has been no direct comparison of SpaceOAR and DuraSeal; however, there does not appear to be any discernible variation in spacing achieved, rectal dosimetry or rectal toxicity. They differ in the half-life, polymerisation time and cost (Table 3). DuraSeal breaks down after 4–6 [30] weeks, compared to 3–6 months [36] for SpaceOAR, and this may result in reduced protection towards the end of the treatment. Ideally the spacing should be present for the entire duration of the treatment, and this is a particular concern in LDR PBT due to the longer duration of the treatment of up to a few months. However, in one study of 10 consecutive patients with DuraSeal, receiving LDR PBT the gel volume clearance had a half-life of 47 days, but the rectal separation half-life was 110 days due to oedema [15]. This suggests that DuraSeal spacer may provide a longer protection than was initially thought. The polymerisation times differ, with DuraSeal rapidly polymerising in 3–4 s [24] compared to SpaceOAR in 10 s [36]. This may lead to an increased likelihood of needle plugging; however, in this review, there was only one case of premature coagulation which occurred in a study using SpaceOAR. DuraSeal polymerisation can be delayed slightly by diluting it 1:1 with saline [24]. The major advantage of DuraSeal over SpaceOAR is the cost which is 4 times lower than SpaceOAR [15].

**Table 3:** Comparison of DuraSeal and SpaceOAR

	DuraSeal	SpaceOAR
Manufacturer	Covidien	Boston Scientific
Approval	Off label (approved for use in spinal surgery) [37]	FDA approval and CE marked [13]
Number of studies in this review using this spacer	4	5
Polymerisation time	4 s [24]	10 s [36]
Spacer half-life	4–6 weeks [15, 20]	3 months [30]
Excretion	Renal [24]	Renal [30]
Cost	£250 (€300) [15]	£1250 (€1500) [15]

### Clinical benefits of PEG spacers

Increasing the space between the rectum and the prostate reduces the rectal dosimetry and a reduction in rectal dosimetry has been shown to reduce adverse events [38]. Dose sparing to the rectum is significant whether the separation achieved is 1 mm or 1 cm [39]. Even a small increase in the space results in risk reduction due to a rapid dose fall off with PBT. Gel spacers seem to increase the mean rectal spacing from 7.7 to 16 mm, with variation possibly caused by the proportion of salvage PBT patients and where the measurement was taken from [14, 20]. The spacers had no difference in their effectiveness in patients with a raised BMI [19]. All studies in this review demonstrated a reduction in rectal dosimetry with no significant differences in bladder or prostate dosimetry [19, 21]. This is in accordance with studies of PEG hydrogel spacers in other radiotherapy modalities [40, 41].

Radiotherapy caused GI symptoms such as diarrhoea and rectal bleeding and both single arm and case–control studies reported lower than usual acute GI toxicity [13, 17, 20]. Chao et al*.* in their retrospective study compared late GI toxicity in a non-spacer and spacer group, and noted a non-significant reduction of late GI toxicity in the spacer group [17]. There have been prospective randomised trials analysing late GI toxicity and sexual function with the use of PEG hydrogel spacers in IMRT. A randomised controlled phase III trial of SpaceOAR for rectal spacing compared with no spacer in IMRT has demonstrated improved quality of life, rectal toxicity and sexual function, with a median follow up of 3 years [41, 42]. At 37 months an improvement in sexual function of baseline impotent men was observed with 37 % of controls and 66.7% of spacer patients capable of achieving erections sufficient for intercourse [40].

### Potential risks of PEG spacers

The PEG hydrogel spacer was generally well tolerated with some studies reporting no adverse effects [19, 21]. However, there are several potential risks (Table 4). Minor complications such as rectal discomfort or tenesmus were noted which settled without intervention [15, 26]. Rectal ulceration was noted in a case report, this may have been caused by infection, mechanical injury, ischaemic injury or radiation injury [25].

**Table 4:** Potential complications from rectal spacer injection

Mild	Moderate	Severe
Sensation of rectal fullness/ pain [15]	Infection, e.g., prostatitis/ rectal abscess [16, 20]	Systemic embolism if air/ gel injected intravenously [43]
Tenesmus [43]	Rectal ulcers [30]	Fistula requiring colostomy/ urostomy [14, 20]
Diarrhoea [20]		Anaphylaxis [43]

A concern was raised by a review of complications of SpaceOAR infections in the Manufacturer and User Facility Device Experience (MAUDE) Database [43]. This review found 25 major complications reported including acute pulmonary embolism, severe anaphylaxis, prostatic abscess and sepsis, purulent perineal drainage, rectal wall erosion and recto-urethral fistula were reported, with surgical intervention required in 11 cases. There are limitations of the MAUDE study including limited data about the patient and disease characteristics and the complications may be related to the disease process or patient co-morbidities rather than the hydrogel spacer or the inserter. Radiotherapy itself may be responsible for many of the complications. The most severe complications in this review of 671 patients were 2 cases (0.30%) of prostatorectal fistulas requiring diverting colostomies [14, 20]. A systematic review of LDR PBT found a similar rate of developing fistulas (0.25%) [44]. Furthermore, one of these cases was a patient receiving salvage PBT. Of the 251 cases of salvage PBT reported in the literature from 1990 to 2007, a higher rate of patients developing fistulas has been reported (3.4%) [45].

### Limitations of the study

Whilst this study provides the most up-to-date review of PEG hydrogel spacers for PBT, this review is limited by the quality of evidence of the studies it is based on. The studies reviewed were all retrospective and non-randomised. Only 4 studies compared their outcomes with controls. Of these only 2 studies compared their complication rate with a control arm. None of the studies comment on cancer outcomes, although no study found a significant difference in radiation dose to the prostate between cases and controls. There is some heterogeneity between the studies both in the treatment method and type of spacer used which prevented a formal meta-analysis from being performed. A larger selection of case–control trials or a randomised control trial, comparing both GI complications and oncological outcomes, is needed.

## Conclusion

PEG hydrogel spacers appear safe to insert. Gel insertion is easy, fast and has a low rate of failure. These studies convincingly demonstrate a significant reduction in rectal dosimetry. Although the results of spacers in reducing rectal toxicity is promising, these need to be confirmed in prospective randomised trial.

## Electronic supplementary material

Below is the link to the electronic supplementary material.

Supplementary file1 (DOCX 18 kb)
