# Supplementary material for: Polyethylene glycol-based hydrogel rectal spacers for prostate brachytherapy: a systematic review with a focus on technique
Source: World J Urol. 2020 Aug 25;39(6):1769–80. doi: 10.1007/s00345-020-03414-6 (PMC8217059; doi:10.1007/s00345-020-03414-6)
Supplement: Supplementary file 1 — Supplementary file1 (DOCX 18 kb) [file 345_2020_3414_MOESM1_ESM.docx]

**Supplemental Online Table:**

Table 6. Responses by gender on questionnaire items.

|  | | **Sex** | | | | | |
| --- | --- | --- | --- | --- | --- | --- | --- |
|  |  | **Male** | | **Female** | | **Total** | |
|  |  | **n** | **%** | **n** | **%** | **N** | **%** |
| **1. Do you apply sunscreen daily to your FACE?** | **Always** | 2 | 7.4% | 28 | 65.1% | 30 | 42.9% |
|  | **Sometimes** | 14 | 51.9% | 9 | 20.9% | 23 | 32.9% |
|  | **Rarely** | 9 | 33.3% | 4 | 9.3% | 13 | 18.6% |
|  | **Never** | 2 | 7.4% | 2 | 4.7% | 4 | 5.7% |
| **2. Do you apply sunscreen daily to your BODY?** | **Always** | 0 | 0.0% | 6 | 14.0% | 6 | 8.6% |
|  | **Sometimes** | 9 | 33.3% | 25 | 58.1% | 34 | 48.6% |
|  | **Rarely** | 9 | 33.3% | 7 | 16.3% | 16 | 22.9% |
|  | **Never** | 9 | 33.3% | 5 | 11.6% | 14 | 20.0% |
| **3. Do you apply sunscreen daily to your FACE and BODY?** | **Always** | 0 | 0.0% | 8 | 18.6% | 8 | 11.4% |
|  | **Sometimes** | 12 | 44.4% | 20 | 46.5% | 32 | 45.7% |
|  | **Rarely** | 11 | 40.7% | 11 | 25.6% | 22 | 31.4% |
|  | **Never** | 4 | 14.8% | 4 | 9.3% | 8 | 11.4% |
| **4. Do you re-apply sunscreen every two hours if you are outdoors?** | **Always** | 0 | 0.0% | 15 | 34.9% | 15 | 21.4% |
|  | **Sometimes** | 7 | 25.9% | 12 | 27.9% | 19 | 27.1% |
|  | **Rarely** | 10 | 37.0% | 11 | 25.6% | 21 | 30.0% |
|  | **Never** | 10 | 37.0% | 5 | 11.6% | 15 | 21.4% |
| **5. Do you regularly wear a hat if you are outdoors?** | **Always** | 14 | 51.9% | 13 | 30.2% | 27 | 38.6% |
|  | **Sometimes** | 8 | 29.6% | 18 | 41.9% | 26 | 37.1% |
|  | **Rarely** | 4 | 14.8% | 9 | 20.9% | 13 | 18.6% |
|  | **Never** | 1 | 3.7% | 3 | 7.0% | 4 | 5.7% |
| **6. Do you regularly wear sunglasses if driving or outdoors?** | **Always** | 12 | 44.4% | 32 | 74.4% | 44 | 62.9% |
|  | **Sometimes** | 4 | 14.8% | 8 | 18.6% | 12 | 17.1% |
|  | **Rarely** | 6 | 22.2% | 3 | 7.0% | 9 | 12.9% |
|  | **Never** | 5 | 18.5% | 0 | 0.0% | 5 | 7.1% |
| **7. How many bottles or tubes of sunscreen do you use per year?** | **1** | 12 | 46.2% | 11 | 25.6% | 23 | 33.3% |
|  | **2** | 2 | 7.7% | 6 | 14.0% | 8 | 11.6% |
|  | **3** | 4 | 15.4% | 11 | 25.6% | 15 | 21.7% |
|  | **4** | 2 | 7.7% | 5 | 11.6% | 7 | 10.1% |
|  | **5** | 2 | 7.7% | 4 | 9.3% | 6 | 8.7% |
|  | **6** | 4 | 15.4% | 3 | 7.0% | 7 | 10.1% |
|  | **7** | 0 | 0.0% | 0 | 0.0% | 0 | 0.0% |
|  | **8** | 0 | 0.0% | 0 | 0.0% | 0 | 0.0% |
|  | **9** | 0 | 0.0% | 1 | 2.3% | 1 | 1.4% |
|  | **10+** | 0 | 0.0% | 2 | 4.7% | 2 | 2.9% |
| **8. Did you get a sunburn this past year?** | **No** | 23 | 85.2% | 33 | 76.7% | 56 | 80.0% |
|  | **Yes** | 4 | 14.8% | 10 | 23.3% | 14 | 20.0% |
| **9. After getting skin cancer, did you use sunscreen?** | **More often** | 22 | 81.5% | 33 | 76.7% | 55 | 78.6% |
|  | **Less often** | 0 | 0.0% | 2 | 4.7% | 2 | 2.9% |
|  | **No change in usage** | 5 | 18.5% | 8 | 18.6% | 13 | 18.6% |
| **10. How often do you get a skin examination in Dermatology?** | **Once per year** | 11 | 40.7% | 15 | 34.9% | 26 | 37.1% |
|  | **Twice per year** | 14 | 51.9% | 23 | 53.5% | 37 | 52.9% |
|  | **More than twice per year** | 2 | 7.4% | 5 | 11.6% | 7 | 10.0% |
| **11. How often do you perform a self-skin examination at home?** | **Never** | 2 | 7.4% | 3 | 7.0% | 5 | 7.1% |
|  | **Once per year** | 3 | 11.1% | 0 | 0.0% | 3 | 4.3% |
|  | **Twice per year** | 4 | 14.8% | 3 | 7.0% | 7 | 10.0% |
|  | **3+ times per year** | 5 | 18.5% | 8 | 18.6% | 13 | 18.6% |
|  | **Monthly** | 13 | 48.1% | 29 | 67.4% | 42 | 60.0% |
| **12. Smoker** | **Current** | 0 | 0.0% | 1 | 2.3% | 1 | 1.4% |
|  | **Past** | 11 | 40.7% | 9 | 20.9% | 20 | 28.6% |
|  | **Never/No** | 16 | 59.3% | 33 | 76.7% | 49 | 70.0% |
| **13. Most non-melanoma skin cancers are related to ultraviolet (sun) exposure.** | **False** | 3 | 11.1% | 6 | 14.0% | 9 | 12.9% |
|  | **True** | 24 | 88.9% | 37 | 86.0% | 61 | 87.1% |
| **14. A base tan is a healthy way to protect your skin from the sun.** | **False** | 23 | 85.2% | 42 | 97.7% | 65 | 92.9% |
|  | **True** | 4 | 14.8% | 1 | 2.3% | 5 | 7.1% |
| **15. Sunburns increase risk of developing melanoma skin cancer.** | **False** | 0 | 0.0% | 1 | 2.3% | 1 | 1.4% |
|  | **True** | 27 | 100.0% | 42 | 97.7% | 69 | 98.6% |
| **16. Regular sunscreen use can reduce risk for skin cancer.** | **False** | 0 | 0.0% | 1 | 2.3% | 1 | 1.4% |
|  | **True** | 27 | 100.0% | 42 | 97.7% | 69 | 98.6% |
